# Smoking and other determinants of bone turnover

**DOI:** 10.1371/journal.pone.0225539

**Published:** 2019-11-25

**Authors:** Rolf Jorde, Astrid Kamilla Stunes, Julia Kubiak, Guri Grimnes, Per Medbøe Thorsby, Unni Syversen

**Affiliations:** 1 Tromsø Endocrine Research Group, Department of Clinical Medicine, UiT, The Arctic University of Norway, Tromsø, Norway; 2 Division of Internal Medicine, University Hospital of North Norway, Tromsø, Norway; 3 Department of Clinical and Molecular Medicine, Faculty of Medicine and Health Sciences, Norwegian University of Science and Technology (NTNU), Trondheim, Norway; 4 Clinic of Medicine, St. Olavs Hospital, Trondheim University Hospital, Trondheim, Norway; 5 Hormone Laboratory, Department of Medical Biochemistry, Oslo University Hospital, Aker Hospital, Oslo, Norway; 6 Department of Endocrinology, Clinic of Medicine, St. Olav’s Hospital, Trondheim University Hospital, Trondheim, Norway; University of Michigan, UNITED STATES

## Abstract

The balance between bone resorption and formation may be assessed by measurement of bone turnover markers (BTMs), like carboxyl-terminal cross-linked telopeptide of type 1 collagen (CTX-1) and procollagen type 1 amino-terminal propeptide (P1NP). Smoking has been shown to influence bone turnover and to reduce bone mass density (BMD), the exact mechanism for this is, however, not settled. In this post-hoc study including 406 subjects (mean age 51.9 years), we aimed to study the impact of smoking on bone turnover. Moreover, we wanted to assess the inter-correlation between substances regulating bone metabolism and BTMs, as well as tracking over time. BMD measurements and serum analyses of CTX-1, P1NP, osteoprotegerin (OPG), receptor activator of nuclear factor ĸB ligand (RANKL), Dickkopf-1 (DKK1), sclerostin, tumor necrosis factor-α (TNF-α), and leptin were performed. Repeated serum measurements were made in 195 subjects after four months. Adjustments were made for sex, age, body mass index (BMI), smoking status, insulin resistance, serum calcium, parathyroid hormone, 25-hydroxyvitamin D and creatinine. Smokers had higher levels of DKK1 and OPG, and lower levels of RANKL, as reflected in lower BTMs and BMD compared to non-smokers. There were strong and predominantly positive inter-correlations between BTMs and the other substances, and there was a high degree of tracking with Spearman’s rho from 0.72 to 0.92 (P < 0.001) between measurements four months apart. In conclusion, smokers exhibited higher levels of DKK1 and OPG and a lower bone turnover than did non-smokers. The strong inter-correlations between the serum parameters illustrate the coupling between bone resorption and formation and crosstalk between cells.

## Introduction

Adult bone undergoes a continuous remodeling with bone resorption by the osteoclasts and bone formation by the osteoblasts, a process that is governed by the osteocytes [1]. The regulation of bone metabolism is complex, and many signaling pathways are involved [2]. The balance between these processes may be assessed by measurement of bone turnover markers (BTMs) in serum and bone mineral density (BMD) [3]. The recommended BTMs for evaluation of resorption and formation, respectively, are carboxyl-terminal cross-linked telopeptide of type 1 collagen (CTX-1), a degradation product of type 1 collagen bone resorption, and procollagen type 1 amino-terminal propeptide (P1NP) [4].

Bone resorption and formation are orchestrated by many substances. Receptor activator of nuclear factor ĸB ligand (RANKL) promotes osteoclastogenesis and bone resorption [5]. Tumor necrosis factor-α (TNF-α) has an important role in inflammation and stimulates bone resorption in synergy with RANKL [6]. Osteoprotegerin (OPG) is a decoy receptor that binds RANKL and thereby inhibits osteoclast formation [5]. Sclerostin and Dickkopf-1 (DKK1) are potent inhibitors of bone formation via blocking the canonical WNT signaling pathway [7]. The multifunctional adipokine leptin stimulates bone formation by a peripheral pathway and appears to inhibit bone formation through a central pathway as well [8]. Moreover, parathyroid hormone (PTH) and vitamin D are crucial in regulation of serum calcium levels, and also have direct effects on bone [9]. Accordingly, measurements of these substances may yield insight into the mechanisms for alterations in serum levels of BTMs.

Several factors may affect bone homeostasis, including smoking and body mass index (BMI) [10, 11]. Smoking is associated with increased risk for osteoporosis, the exact mechanisms are, however, not settled [12]. We recently performed a vitamin D RCT on cardiovascular risk factors and BTMs in a large group of subjects [13]. Supplementation with vitamin D for four months had minor effects on CTX-1 and P1NP and the other parameters mentioned above [14]. In the present study we did a post-hoc study of this population, addressing the impact of smoking and other factors on bone turnover. Moreover, we examined the inter-correlations between regulators of bone homeostasis and BTMs and their tracking over time.

## Methods

### Subjects and study design

The design of the study has previously been described in detail [13]. In short, the study was performed in Tromsø, northern Norway (69 degrees north). The subjects were recruited from the population based Tromsø study [15] where 1489 subjects with serum 25-hydroxyvitamin D (25(OH)D) < 42 nmol/L and age < 80 years were invited, 455 subjects came to a screening visit, and 422 subjects were included and randomized to vitamin D versus placebo for four months. Exclusion criteria were granulomatous disease, diabetes, renal stones last five years, or serious diseases that would make the subject unfit for participation, use of vitamin D supplements exceeding 800 IU vitamin D per day, use of solarium on a regular basis, and planned holiday(s) in tropical areas during the intervention period. Women of childbearing potential without use of acceptable contraception (hormonal, IUD) were not included.

All subjects not using anti-resorptive treatment and with successful measurements of BTMs at baseline were included in the cross-sectional BTM analyses, and subjects in the placebo group with successful measurements on both occasions, were included in the tracking analyses.

### Measurements

The same measurements were performed at baseline and after four months. Height and weight were measured wearing light clothing and no shoes, and fasting blood samples were drawn. Serum calcium and creatinine, PTH, 25(OH)D, blood glucose, serum insulin and HbA_1c_ and homeostatic model assessment for insulin resistance (HOMA-IR) were calculated as previously described [13]. BMI was calculated as weight (kg) divide by height (m) squared.

CTX-1 and P1NP were measured by electrochemiluminescence immunoassays with a Cobas e601 kit (Roche Diagnostics, NJ, USA), at the Hormone Laboratory, Oslo University Hospital, Norway. DKK1, leptin, OPG, sclerostin, TNF-α were analyzed using multianalyte profiling Milliplex MAP assay, and RANKL by a single analyte assay (Millipore Corporation, Billerica, MA, USA).

BMD was measured by DXA (GE Lunar Prodigy, Lunar Corporation, Madison, WI, USA) at the hip and lumbar spine, with total hip (mean of left and right, or one side if not both could be measured) and L1 (which had valid measurement in almost every subject) used in the analyses.

### Statistical analyses

Normal distribution was evaluated with skewness (between -1 and 1) and kurtosis (between—3 and 3) and visual inspection of histograms and found normal for all dependent parameters except CTX-1, leptin, OPG and sclerostin that attained normal distribution after logarithmic transformation (log10) and used as such in the regression analyses. Where logarithmically transformed the variables are given the prefix”lg.”. RANKL was not normally distributed and could not be log transformed and was analyzed with non-parametric statistics. Comparisons between groups at baseline were performed with the Student´s t-test or the Mann-Whitney U test. Correlations were evaluated with partial correlations coefficients with control variables or with Spearman´s rho. Linear regression models were used for evaluation of predictors for the BTMs and the other substances. Sex, age, BMI and smoking status were forced into the model with serum calcium, creatinine, PTH, 25(OH)D and HOMA as potential significant covariates using the stepping method with entry criteria of 0.05 and removal criteria 0.10. Because of the high n, the regression line is indicated in the figures also for non-parametric correlations. There were no observations with extreme leverage.

P < 0.05 (two-tailed) was considered statistically significant. Data are presented as mean ± SD or as median (5, 95 percentile). All statistical analyses were performed using IBM SPSS version 22 software.

### Ethics

The study was approved by the Regional Committee for Medical Research Ethics (REK NORD 2013/1464) and by the Norwegian Medicines Agency (2013-003514-40). The study is registered at ClinicalTrials.gov NCT02750293. All subjects gave their written informed consent.

## Results

Four-hundred and six subjects had successful measurements of BTMs at baseline and were included in the cross-sectional analyses. Their characteristics are shown in Tables 1 and 2 in relation to gender and smoking status. Males had significantly higher BMD at the total hip, higher serum TNF-α and sclerostin, and significantly lower serum leptin than females. These relations to sex were not dependent on age and were also seen in stratified analyses (age < 45, age 45–55, > 55 years, S1 Table). Smokers had significantly lower BMD at the total hip, lower P1NP, CTX-1 and RANKL, and higher DKK1 and OPG than non-smokers. Smokers also had significantly lower creatinine than non-smokers, a difference that also was significant (P < 0.001) after adjusting for sex, age and BMI.

**Table 1:** Characteristics of the subjects at baseline and in relation to gender.

	All subjects (n = 406)	Males (n = 212)	Females (n = 194)	Mean difference (95% CI)	P-value*
Males/females	212/194				
Current smokers/non-smokers	86/320	47/165	39/155		0.629
Age (years)	51.9 ± 8.7	52.0 ± 9.0	51.6 ± 8.3	-0.4 (-2.1, 1.3)	0.644
BMI (kg/m^2^)	27.8 ± 4.9	28.1 ± 4.6	27.4 ± 5.3	-0.6 (-1.6, 0.3)	0.191
Serum calcium (mmol/L)	2.27 ± 0.07	2.26 ± 0.007	2,26 ± 0.08	-0.02 (-0.03, -0.01)	0.008
Serum creatinine (μmol/L)	71.3 ± 12.4	77.9 ± 11.6	64.1 ± 8.6	-13.8 (-15.8, -11.8)	<0.001
Serum PTH (pmol/L)	6.7 ± 2.0	6.6 ± 1.9	6.9 ± 2.2	0.3 (-0.1, 0.7)	0.115
Serum 25(OH)D (nmol/L)	34.0 ± 12.9	33.9 ± 13.2	34.1 ± 12.5	0.2 (-2.3, 2.7)	0.862
HbA1c (%)	5.49 ± 0.31	5.15 ± 0.33	5.47 ± 0.29	-0.04 (-0.10, 0.03	0.257
HOMA-IR	2.72 (0.94, 8.28)	3.38 (1.08, 11.84)	2.14 (0.84, 6.32)		0.000
Serum P1NP (pg/ml)	44.8 ± 15.1	44.5 ± 13.7	45.2 ± 16.6	0.7 (-2.2, 3.7)	0.625
Serum CTX-1 (pg/ml)	0.34 (0.18, 0.62)	0.36 (0.19, 0.67)	0.35 (0.16, 0.59)		0.207
Serum DKK1 (pg/ml)	1456 ± 396	1461 ± 402	1451 ± 392	-10 (-87, 68)	0.808
Serum Leptin (pg/ml)	11081 (1725, 53375)	7488 (1212, 30290)	20094 (3051, 68867)		<0.001
Serum TNF-α (pg/ml)	2.40 ± 0.81	2.58 ± 0.82	2.20 ± 0.76	-0.38 (-0.53, -0.22)	<0.001
Serum OPG (pg/ml)	306 (192, 479)	306 (188, 498)	306 (208, 460)		0.979
Serum sclerostin (pg/ml)	1806 (1030, 3140)	2044 (1126, 3286)	1642 (998, 2764)		<0.001
Serum RANKL (pg/ml)	0.0 (0.0, 46.8)	0.0 (0.0, 55.8)	0.0 (0.0, 24.9)		0.002
BMD total hip (g/cm^2^)**	0.993 ± 0.133	1.032 ± 0.118	0.950 ± 0.136	-0.081 (-0.109, -0.054)	0.000
BMD L1 (g/cm^2^)**	1.067 ± 0.156	1.084 ± 0.159	1.048 ± 0152	-0.036 (-0.070, -0.003)	0.035

* Females vs males (Chi-square test, student’s t-test or Mann-Whitney U test)

** 334 subjects (177 males, 157 females; 265 non-smokers, 69 smokers)

Data shown as mean ± SD or median (5, 95 percentile)

**Table 2:** Characteristics of the subjects at baseline in relation to smoking status.

	Non-smokers (n = 320)	Smokers (n = 86)	Mean difference (95% CI)	P-value*
Males/females	165/155	47/39		0.629
Age (years)	51.6 ± 8.6	52.7 ± 8.7	-1.0 (-3.1, 1.0)	0.326
BMI (kg/m^2^)	28.0 ± 4.9	27.1 ± 4.8	0.8 (-0.3, 2.0)	0.156
Serum calcium (mmol/L)	2.27 ± 0.07	2.28 ± 0.08	-0.01 (-0.03, 0.01)	0.329
Serum creatinine (μmol/L)	72.7 ± 12.1	66.2 ± 12.0	6.5 (3.6, 9.4)	<0.001
Serum PTH (pmol/L)	6.9 ± 2.0	6.2 ± 2.0	0.7 (0.2, 1.2)	0.005
Serum 25(OH)D (nmol/L)	34.8 ± 13.0	31.0 ± 12.0	3.8 (0.7, 6.8)	0.013
HbA1c (%)	5.46 ± 0.31	5.61 ± 0.30	-0.16 (-0.23, -0.08)	<0.001
HOMA-IR	2.79 (0.96, 8.36)	2.62 (0.80, 7.30)		0.191
Serum P1NP (pg/ml)	45.8 ± 15.5	41.1 ± 12.9	4.7 (1.1, 8.3)	0.010
Serum CTX-1 (pg/ml)	0.36 (0.19, 0.63)	0.31 (0.17, 0.56)		0.006
Serum DKK1 (pg/ml)	1435 ± 391	1537 ± 410	-102 (-196, -8)	0.034
Serum Leptin (pg/ml)	11189 (1796, 59548)	9328 (1320, 37599)		0.051
Serum TNF-α (pg/ml)	2.39 ± 0.81	2.45 ± 0.83	-0.06 (-0.26, 0.13)	0.520
Serum OPG (pg/ml)	303 (191, 459)	325 (208, 525)		0.020
Serum sclerostin (pg/ml)	1852 (1016, 3140)	1768 (1126, 2936)		0.895
Serum RANKL (pg/ml)	0.0 (0.0, 50.3)	0.0 (0.0, 22.1)		0.046
BMD total hip (g/cm^2^)**	1.001 ± 0.136	0.964 ± 0.119	0.037 (0.002, 0.073)	0.037
BMD L1 (g/cm^2^)**	1.073 ± 0.150	1.041 ± 0.178	0.032 (-0.009, 0.074)	0.129

*Non-smokers vs smokers (Chi-square test, student’s t-test or Mann-Whitney U test)

** 334 subjects (177 males, 157 females; 265 non-smokers, 69 smokers)

Data shown as mean ± SD or median (5, 95 percentile)

### Determinants of the BTMs and bone regulating substances

In the linear regression model with sex, age, BMI, smoking status, serum calcium, creatinine, PTH, 25(OH)D and HOMA-IR as potential confounders, the above relations between BTMs and bone regulating substances, sex and smoking status were confirmed (Tables 3 and 4). Age was strongly associated with OPG and sclerostin, but not with P1NP or CTX-1. The age-OPG and age-sclerostin relations were seen in both genders, for OPG only in those above 50 years of age, and for sclerostin in subjects both above and below 50 years of age (S2 Table).

**Table 3:** Beta coefficients with 95% confidence intervals from linear regression models for bone turnover markers with sex, age, BMI and smoking status as variables forced into the model, and with serum calcium, creatinine, PTH, 25(OH)D and HOMA as potential significant covariates in the 406 subjects.

	P1NP		Lg. CTX-1		DKK1		Lg. Leptin	
	ß (95% CI)	P-value	ß (95% CI)	P-value	ß (95% CI)	P-value	ß (95% CI)	P-value
Sex*	-0.432 (-3.363, 2.499)	0.772	-0.006 (-0.045, 0.033)	0.764	-14.37 (-90.42, 61.68)	0.711	-0.478 (-0.534, -0.423)	<0.001
Age (years)	0.132 (-0.038, 0.301)	0.127	0.001 (0.000, 0.003)	0.126	-4.587 (-8.953, -0.221)	0.040	-0.001 (-0.004, 0.002)	0.435
BMI (kg/m^2^)	-0.327 (-0.627, -0.026)	0.033	-0.008 (-0.011, -0.004)	<0.001	16.03 (8.302, 23.75)	<0.001	0.050 (0.043, 0.057)	<0.001
Smoking status**	-5.092 (-8.675, -1.509)	0.005	-0.044 (-0.085, -0.004)	0.032	114.8 (22.55, 207.0)	0.015	-0.029–0.093, 0.036)	0.379
Serum creatinine (μmol/L)			0.003 (0.001, 0.004)	0.002				
Serum PTH (pmol/L)			0.010 (0.002, 0.018)	0.020			0.019 (0.006, 0.033)	0.005
Serum calcium (mmol/L)			0.264 (0.041, 0.486)	0.020	653.5 (132.6, 1174)	0.014		
Serum 25(OH)D (nmol/L)								
HOMA-IR							0.034 (0.021, 0.046)	<0.001
Adjusted R^2^	0.035		0.134		0.075		0.672	

*Males = 1. females = 0;

**smokers = 1. non-smokers = 0

**Table 4:** Beta coefficients with 95% confidence intervals from linear regression models for bone turnover markers with sex, age, BMI and smoking status as variables forced into the model, and with serum calcium, creatinine, PTH, 25(OH)D and HOMA as potential significant covariates in the 406 subjects.

	TNF-α		Lg. OPG		Lg. sclerostin		RANKL	
	ß (95% CI)	P-value	ß (95% CI)	P-value	ß (95% CI)	P-value	Spearman´s rho	P-value
Sex*	0.035 (0.144, 0.465)	<0.001	-0.012 (-0.035, 0.010)	0.286	0.064 (0.031, 0.096)	<0.001		
Age (years)	0.001 (-0.008, 0.010)	0.837	0.006 (0.005, 0.007)	<0.001	0.006 (0.004, 0.007)	<0.001	-0.102	0.040
BMI (kg/m^2^)	0.014 (-0.006, 0.034)	0.176	-0.002 (-0.005, 0.001)	0.237	0.004 (0.001, 0.006)	0.010	0.135	0.006
Smoking status**	0.085 (-0.102, 0.272)	0.370	0.032 (0.005, 0.058)	0.018	0.003 (-0.031, 0.036)	0.875		
Serum creatinine (μmol/L)					0.002 (0.001, 0.003)	0.004	0.107	0.030
Serum PTH (pmol/L)					-0.010 (-0.017, -0.003)	0.004	-0.043	0.385
Serum calcium (mmol/L)					-0.232 (-0.416, -0.049)	0.013	-0.009	0.858
Serum 25(OH)D (nmol/L)							-0.100	0.044
HOMA-IR	0.043 (0.005, 0.081)	0.027	0.007 (0.002, 0.013)	0.006			0.148	0.003
Adjusted R^2^	0.095		0.216		0.238			

*Males = 1. females = 0;

**smokers = 1. non-smokers = 0

BMI was negatively associated with CTX-1, and positively associated with the bone formation inhibitor DKK1. Serum creatinine was positively associated to CTX-1 and sclerostin, whereas PTH and serum calcium showed only few weak associations. Remarkably, there were no significant associations between 25(OH)D and the BTMs or bone regulating substances in the linear regression model. Furthermore, there were significant relations for HOMA-IR with leptin and OPG (Tables 3 and 4).

Except for leptin, where the regression model had an adjusted R^2^ of 0.666, the other regression models only explained 4–23% of the variance of the BTMs or bone regulating substances. In particular, the R^2^ for P1NP and CTX-1 were 0.035 and 0.118, respectively. Inclusion of the bone regulating substances DKK1, leptin, TNF-α, OPG, sclerostin and RANKL as co-variates in the model increased the R^2^ to 0.062 and 0.149 for P1NP and CTX-1, respectively.

### Correlations between the BTMs

**Fig 1:**
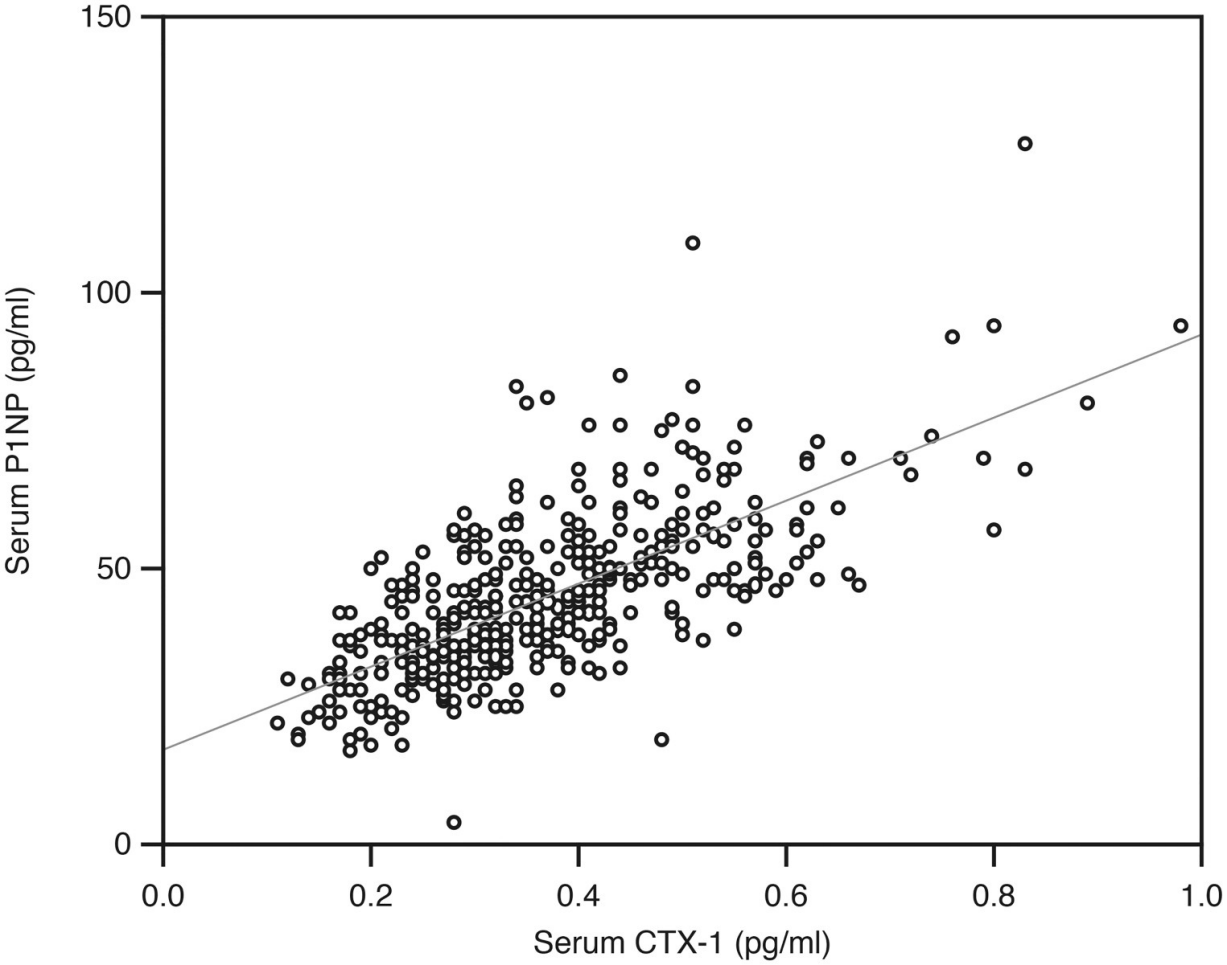
Relation between the serum CTX-1 and P1NP in the 406 subjects.

In the analyses with partial correlations controlling for sex, age, BMI, smoking status, serum calcium, creatinine, PTH, 25(OH)D and HOMA-IR, there was a strong positive correlation between P1NP and CTX-1 (r = 0.67, P < 0.001) (Fig 1), and also several significant associations in-between the bone regulating substances. All of these associations were positive, except for a weak negative association between sclerostin and CTX-1 (Tables 5 and 6).

**Table 5:** Partial correlation coefficients for bone turnover markers with sex, age, BMI, smoking status, serum calcium, creatinine, PTH, 25(OH)D and HOMA as control variables in the 406 subjects.

	P1NP		Lg. CTX-1		DKK1		Lg. Leptin	
	Partial correlation coefficient	P-value	Partial correlation coefficient	P-value	Partial correlation coefficient	P-value	Partial correlation coefficient	P-value
Serum P1NP (pg/ml)			0.655	<0.001	0.014	0.774	-0.012	0.813
Lg. serum CTX-1 (pg/ml)	0.655	<0.001			0.001	0.982	-0.043	0.395
Serum DKK1 (pg/ml)	0.014	0.774	0.001	0.982			0.205	<0.001
Lg. serum Leptin (pg/ml)	-0.012	0.813	-0.043	0.395	0.205	<0.001		
Serum TNF-α (pg/ml)	0.104	0.040	0.147	0.003	0.237	<0.001	0.150	0.003
Lg. serum OPG (pg/ml)	0.102	0.043	-0.017	0.735	0.199	<0.001	0.160	0.001
Lg. serum sclerostin pg/ml)	-0.084	0.095	-0.120	0.017	0.139	0.006	0.138	0.006
BMD total hip (g/cm^2^)*	-0.208	<0.001	-0.224	<0.001	0.039	0.481	-0.026	0.640
BMD L1 (g/cm^2^)*	-0.116	0.038	-0.143	0.010	-0.007	0.907	0.054	0.038

*n = 334

**Table 6:** Partial correlation coefficients for bone turnover markers with sex, age, BMI, smoking status, serum calcium, creatinine, PTH, 25(OH)D and HOMA as control variables in the 406 subjects.

	TNF-α		Lg. OPG		Lg. sclerostin		RANKL	
	Partial correlation coefficient	P-value	Partial correlation coefficient	P-value	Partial correlation coefficient	P-value	Spearman´s rho	P-value
Serum P1NP (pg/ml)	0.104	0.040	0.102	0.043	-0.084	0.095	0.013	0.796
Lg. serum CTX-1 (pg/ml)	0.147	0.003	-0.017	0.735	-0.120	0.017	0.040	0.420
Serum DKK1 (pg/ml)	0.237	<0.001	0.199	<0.001	0.139	0.006	0.001	0.978
Lg. serum Leptin (pg/ml)	0.150	0.003	0.160	0.001	0.138	0.006	0.060	0.226
Serum TNF-α (pg/ml)			0.207	<0.001	0.154	0.002	0.223	<0.001
Lg. serum OPG (pg/ml)	0.207	<0.001			0.287	<0.001	-0.141	0.004
Lg. serum sclerostin pg/ml)	0.154	0.002	0.287	<0.001			0.107	0.032
BMD total hip (g/cm^2^)*	-0.026	0.643	0.003	0.964	0.163	0.003	0.072	0.188
BMD L1 (g/cm^2^)*	0.019	0.738	0.084	0.134	0.163	0.003	-0.035	0.528

*n = 334

**Fig 2:**
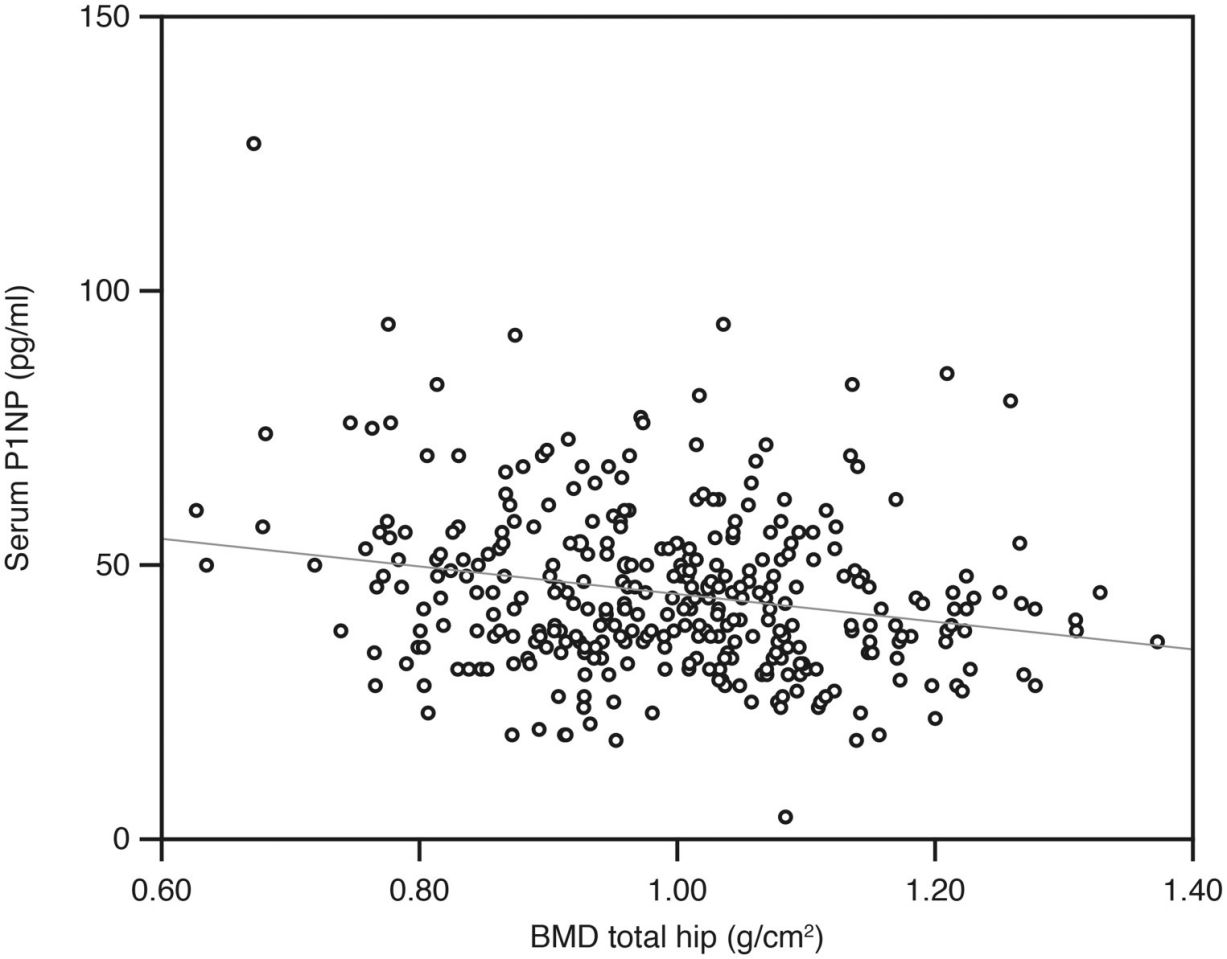
Relation between P1PN and BMD total hip in the 406 subjects.

**Fig 3:**
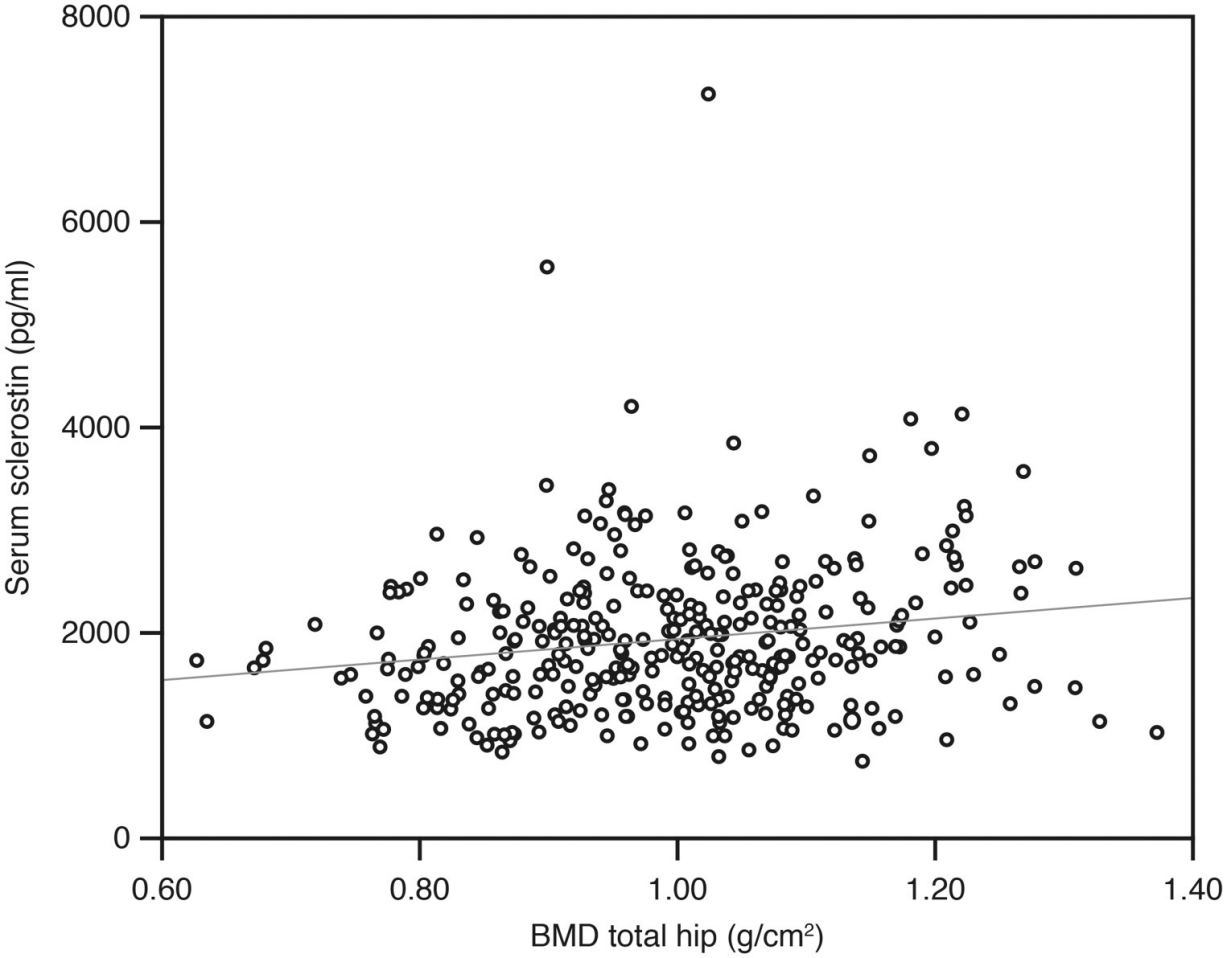
Relation between sclerostin and BMD total hip in the 406 subjects.

There were significant negative correlations between P1NP and CTX-1 versus BMD both at the total hip and L1, shown for P1NP and total hip in Fig 2. For the other bone regulating substances, the only significant association with BMD was a positive correlation with sclerostin (Tables 5 and 6, Fig 3).

### Tracking

**Fig 4:**
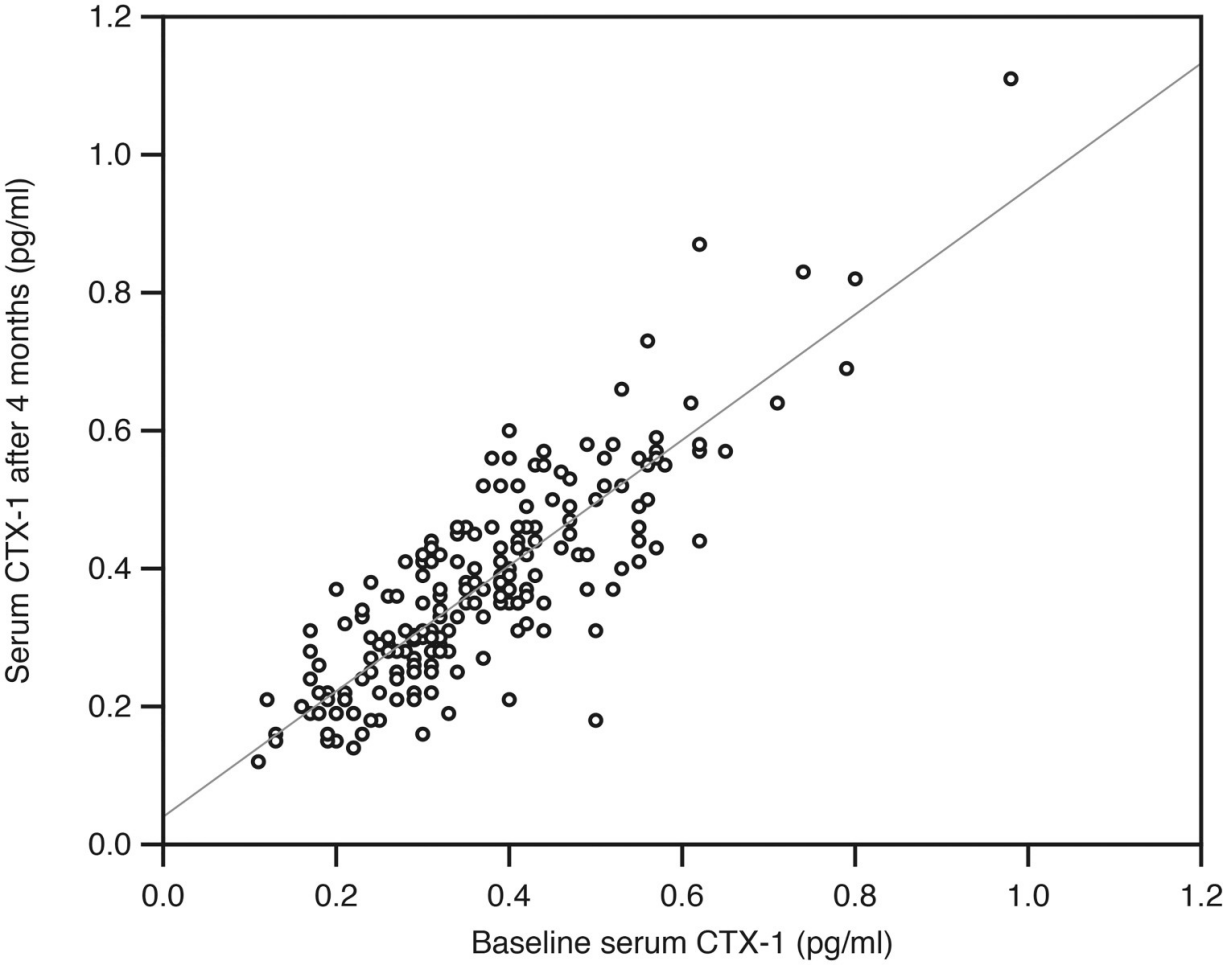
Relation between serum CTX-1 at baseline and after four months in the 195 subjects in the placebo group in the intervention study.

**Fig 5:**
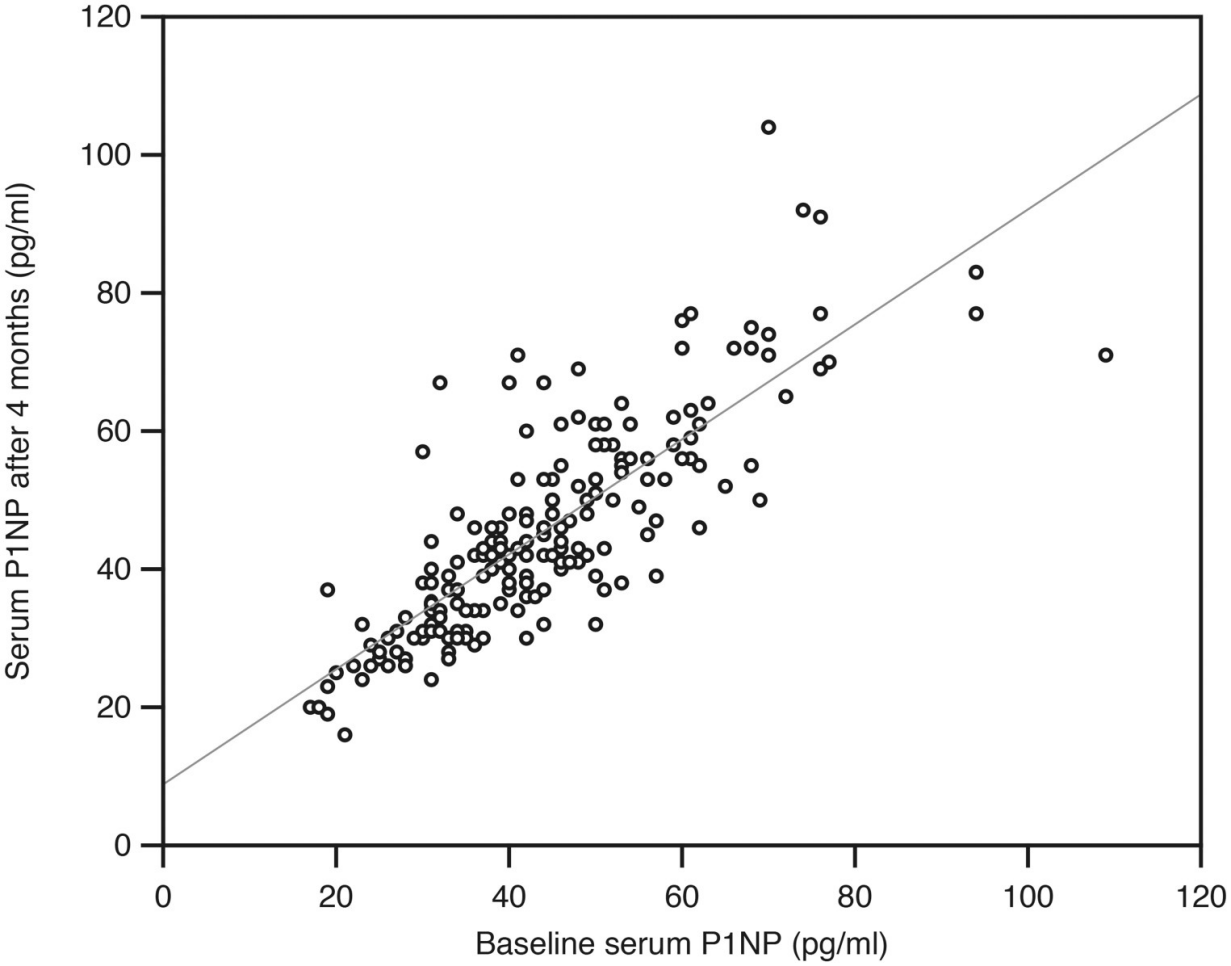
Relation between serum P1NP at baseline and after four months in the 195 subjects in the placebo group in the intervention study.

One hundred and ninety-five subjects in the placebo group completed the four months intervention, had successful BTM measurements at baseline and end of study, and were included in the tracking analyses. Except for RANKL, there was a high degree of tracking from baseline to end of study, with correlation coefficient rho ranging from 0.72 to 0.92 (Figs 4 and 5). These correlations were considerably higher than the corresponding ones for serum calcium, PTH and 25(OH)D (Table 7).

**Table 7:** Spearman’s correlation coefficient rho between serum values at baseline and end of study in the 195 subjects in the placebo group in the intervention study.

Bone turnover marker	Rho between baseline and end of study value	P-value
Serum P1NP	0.821	<0.001
Serum CTX-1	0.819	<0.001
Serum DKK1	0.860	<0.001
Serum Leptin	0.919	<0.001
Serum TNF-α	0.715	<0.001
Serum OPG	0.781	<0.001
Serum sclerostin	0.834	<0.001
Serum RANKL	0.465	<0.001
Serum calcium	0.614	<0.001
Serum creatinine	0.925	<0.001
Serum PTH	0.714	<0.001
Serum 25(OH)D	0.453	<0.001
HOMA-IR	0.760	<0.001

## Discussion

The present study provides novel insight into the mechanisms for the smoking-induced bone loss. Smokers exhibited higher levels of DKK1, reflected in lower level of the bone formation marker P1NP compared to non-smokers. Moreover, OPG, RANKL and PTH levels were lower in smokers, as mirrored in the attenuated level of the bone resorption marker CTX-1. Accordingly, the inferior BMD in smokers may be attributed to a lower bone turnover. To our knowledge, this is the first study to demonstrate enhanced levels of DKK1 and OPG in smokers. A positive correlation was observed between sclerostin levels and BMD. Accordingly, smokers displayed lower sclerostin levels than non-smokers, however, not significant. BMI was negatively related with the BTMs, and positively associated with leptin.

In line with previous studies, we observed lower BMD in smokers compared to non-smokers [12]. This complies with the higher DKK1 levels in smokers causing inhibition of bone formation, as reflected in lower P1NP levels. Sclerostin, another inhibitor of bone formation, tended to be reduced among smokers, reflecting the lower BMD. Our findings concerning the positive relation between circulating sclerostin and BMD support observations in other populations [16] and could be attributed to a larger pool of osteocytes in those with high BMD. In accordance with a study by Reseland *et al*. [17], the smokers displayed lower leptin levels, although not significant, that could contribute to impairment of bone formation.

Bone resorption assessed by CTX-1 was also lower in smokers, which concords with lower levels of RANKL and higher levels of OPG. These findings support most studies [12], with the exception of OPG showing decreased or equal levels compared to non-smokers. The reason for this discrepancy is unclear. It should be kept in mind that circulating OPG may be derived from other sources than bone [18]. In line with several studies, PTH levels were attenuated among smokers, in spite of lower 25(OH)D levels than in non-smokers [19]. The lower PTH levels could contribute to the decline in RANKL and increase in OPG.

Our data indicate that the bone impairment in smokers may be attributed to a lower bone turnover state. Low bone turnover is also observed in patients with type 2 diabetes [20] where a significant increase in fracture risk is seen, in spite of normal or high BMD [21]. Correspondingly, a meta-analysis by Kanis et al. reported an increase in fracture risk among smokers that was substantially greater than that explained by measurement of BMD [22]. Thus, in a low bone turnover state, impairment of bone quality seems to be proportionally more pronounced than the decline in BMD [23].

The pathophysiological mechanisms by which smoking may affect bone are multiple. Smoking induces alterations in calciotropic hormones, has an impact on the pituitary-adrenal axis and sex hormones, has pronounced inflammatory effects and induces oxidative stress [12]. Tobacco smoke contains more than 7000 substances that could contribute to the skeletal effects. The most abundant agent nicotine has been shown to affect both bone formation and resorption. Nicotine inhibits osteogenesis directly through binding to nicotinic acetylcholine receptors on osteoblasts and indirectly by inducing a rise in ACTH and cortisol levels [24]. Excess cortisol inhibits bone formation, and this may be mediated by DKK1 as glucocorticoids have been shown to stimulate DKK1 in vitro [25]. This complies with the enhanced levels of DKK1 among smokers in the present study. Nicotine has also been shown to suppress formation of osteoclasts with large nuclei and reduce the area of resorption, compatible with a suppression of bone resorption [26].

Other constituents of tobacco smoke that may be negative for the skeleton are polycyclic aryl hydrocarbon compounds which have been shown to exert antiosteogenic effects [27]. Whether this occurs via stimulation of DKK1 remains to be explored. One of these substances, benzo[a]pyrene (BaP), which is present in high concentrations in cigarette smoke, has been found to inhibit osteoclast differentiation and bone resorption, probably attributed to a crosstalk between the aryl hydrocarbon receptor and RANKL signaling pathways [28].

Tobacco smoke also contains many heavy metals including cadmium and lead of which bone is one of the main targets [29]. Both cadmium and lead exposure have been observed to inhibit osteoblast differentiation and to increase bone resorption, and are associated with low BMD and increased fracture risk [30]. Exposure of these metals are also shown to affect the calciotropic hormones by reducing vitamin D and PTH levels [30, 31]. A decline in magnesium levels has been reported in subjects exposed to cadmium [31], and in smokers compared with controls [32]. Hypomagnesemia may thus contribute to the lower PTH levels observed in smokers.

Taken together, the different components of tobacco smoke seem to induce effects on both bone formation and resorption that are predominantly inhibitory, resulting in a lower bone turnover than in non-smokers. Our findings are in support of these data and give some additional insight into mechanisms for bone impairment in smokers.

We observed lower serum creatinine in smokers compared to non-smokers as also shown in previous studies [33]. This could be attributed to lower muscle mass among smokers, as demonstrated in several studies [34]. Unfortunately, we do not have data on muscle mass in our study population. The attenuated creatinine could also be ascribed to hyperfiltration as elaborated on by Halimi et el. [35].

The negative associations between P1NP and CTX-1 and BMI are consistent with a decline in bone turnover with increasing weight. This concords with the low bone turnover reported in individuals with metabolic syndrome and type 2 diabetes [36]. Altered adipokine secretion and insulin resistance are some of the factors suggested to explain this relationship [11]. We did, however, not find any relation between insulin resistance, as evaluated by HOMA-IR, and BMD or the formation/resorption markers P1NP and CTX-1. On the other hand, significant correlations were revealed between HOMA-IR and OPG and RANKL. This is in line with previous studies showing a relation between OPG and insulin resistance [37, 38], as well as between RANKL and insulin resistance [39]. In this regard, it should be recalled that these substances are not only produced in bone, as the cardiovascular system and the immune system being the main sources of OPG and RANKL [18], respectively. As shown previously, leptin and TNF-α were also highly correlated with HOMA-IR [40, 41]. Our findings underscore the interplay between the skeleton and energy metabolism, exemplified by *in vitro* studies showing interaction between insulin and osteoblasts [42], as well as proliferation of pancreatic *β*-cells by osteocalcin stimulation [43].

The effect of sex on leptin, TNF-α and sclerostin levels was unrelated to age both in linear regression and in age-stratified analyses, in accordance with other studies [44–46]. The association between sex and leptin was strongly modified after correction for BMI and might have been further reduced if data on fat mass had been available and included in the analyses [47]. Similarly, the strong relations between age and sclerostin and OPG have been reported before [44–46, 48, 49].

As anticipated, there were strong inter-correlations between the substances regulating bone metabolism and the BTMs. This was particularly seen between CTX-1 and P1NP, demonstrating the coupling between bone resorption and formation [6]. Furthermore, almost all correlations between the other substances and BTMs, regardless of assumed effect being promoting or inhibiting bone formation, were positive. This again illustrates the cross-talk between the bone cells.

Tracking is a result of both natural biological variation over time, which includes circadian variation [50] and meal responses [51], as well as assay reproducibility [52]. There is a high degree of tracking for BMD [53] and it was therefore reasonable to assume that this was the case for the bone-active substances and BTMs, which was also found. To our knowledge there are only a few reports on tracking of individual bone related substances [54, 55], but none where these substances and the BTMs are evaluated together. This high degree of tracking makes it likely that our cross-sectional results are valid for bone metabolism over time and not only represent findings from a single measurement.

Our study has several weaknesses. Unfortunately, RANKL levels were below the detection level in a substantial number of the participants, as also reported by others [56–58]. Non-measureable levels were observed primarily in smokers, reflecting the low RANKL levels in this group. We had no information on physical activity and intake of calcium and magnesium, which could affect the bone turnover as well as the BMD [44]. The study was observational, and no conclusions about causality can be drawn. We included mainly subjects with low serum 25(OH)D levels, and although they otherwise were healthy, our results may not be applicable to subjects with vitamin D sufficiency. However, except for RANKL, we found no relations between serum 25(OH)D and the BTMs and bone-related substances, and inclusion of 25(OH)D in the regression model did not affect the results. Furthermore, the regression model only explained less than 15% of the variance of P1NP and CTX-1, even after inclusion of the bone regulating substances in the model. On the other hand, our study has strengths as we included a large group of subjects and measured both BTMs and several other bone regulating substances.

In conclusion, our study gives novel insight into mechanisms for the smoking-induced osteoporosis. The strong inter-correlations between the serum parameters illustrate the coupling between bone resorption and formation and crosstalk between cells. Moreover, the high degree of tracking illustrates the validity of our data.

## Supporting information

S1 Table Supplementary Table 1. Serum leptin, TNF- α, sclerostin and BMD total hip in relation to age and gender.
(DOCX)

S2 Table Supplementary Table 2. Standardized beta coefficients from linear regression models for lg.OPG and lg. sclerostin in relation to sex and age group with sex, age, BMI, smoking status, serum calcium, creatinine, PTH, 25(OH)D and HOMA as covariates in the 406 subjects.
(DOCX)

S1 Data The study’s underlying data set. (SAV)
