# Supplementary material for: Smoking and other determinants of bone turnover
Source: PLoS One. 2019 Nov 25;14(11):e0225539. doi: 10.1371/journal.pone.0225539 (PMC6876776; doi:10.1371/journal.pone.0225539)
Supplement: S1 Table — Serum leptin, TNF- α, sclerostin and BMD total hip in relation to age and gender. (DOCX) [file pone.0225539.s001.docx]

**S1 Table. Serum leptin, TNF- α, sclerostin and BMD total hip in relation to age and gender**

|  |  | Age < 45 years | | Age 45 – 55 years | | Age > 55 years | |
| --- | --- | --- | --- | --- | --- | --- | --- |
|  |  | ___________________________ | | ___________________________ | | ___________________________ | |
|  |  | Males (n = 46) | Females (n=47) | Males (n = 97) | Females (n = 83) | Males (n = 69) | Females (N = 64) |

| Serum Leptin (pg/ml) | 7957 (1212, 43732)* | 21477 (2352, 68868) | 7942 (1143, 32344)* | 19385(3242, 65056) | 5845 (1251, 23027)* | 18362 (4455, 64601) |
| --- | --- | --- | --- | --- | --- | --- |
| Serum TNF-α (pg/ml) | 2.60 (0.85)* | 2.02 (0.69) | 2.64 (0.79)* | 2.23 (0.71) | 2.49 (0.84) | 2.30 (0.86) |
| Serum sclerostin (pg/ml) | 1796 (998, 2688)* | 1383 (861, 2105) | 1894 (1101, 3064)* | 1671 (960, 2820) | 2464 (1480, 3849)* | 1803 (1092, 2724) |
| BMD total hip (g/cm^2^)** | 1.042 (0.116) | 1.009 (0.115 | 1.041 (0.111)* | 0.978 (0.133) | 1.011 (0.130)* | 0.883 (0.125) |

*P < 0.01 vs females (student’s t-test or Mann-Whitney U test)

** 334 subjects (177 males, 157 females)

Data shown as mean ± SD or median (5, 95 percentile)
