# Supplementary material for: Smoking and other determinants of bone turnover
Source: PLoS One. 2019 Nov 25;14(11):e0225539. doi: 10.1371/journal.pone.0225539 (PMC6876776; doi:10.1371/journal.pone.0225539)
Supplement: S2 Table — Standardized beta coefficients from linear regression models for lg.OPG and lg. sclerostin in relation to sex and age group with sex, age, BMI, smoking status, serum calcium, creatinine, PTH, 25(OH)D and HOMA as covariates in the 406 subjects. (DOCX) [file pone.0225539.s002.docx]

**S2 Table. Standardized beta coefficients from linear regression models for lg.OPG and lg. sclerostin in relation to sex and age group with sex, age, BMI, smoking status, serum calcium, creatinine, PTH, 25(OH)D and HOMA as covariates in the 406 subjects**

|  | Lg. OPG | | | | Lg. sclerostin | | | |
| --- | --- | --- | --- | --- | --- | --- | --- | --- |
|  | __________________________________________________ | | | | _________________________________________________ | | | |
|  | Sex | | Age group | | Sex | | Age group | |
|  | ________________________ | | ________________________ | | ________________________ | | _______________________ | |
|  | Males (n = 212) | Females (n = 194) | < 50 years (n = 188) | > 50 years (n = 218) | Males (n = 212) | Females (n = 194) | < 50 years (n = 188) | > 50 years (n = 218) |

| Age (years) | 0.447** | 0.445** | 0.043 | 0.419** | 0.421** | 0.319** | 0.153* | 0.220 |
| --- | --- | --- | --- | --- | --- | --- | --- | --- |
| Adjusted R^2^ | 0.258 | 0.156 | 0.051 | 0.165 | 0.215 | 0.108 | 0.099 | 0.239 |

*P < 0.05. **P < 0.001
